# Dying at home for people experiencing financial hardship and deprivation: How health and social care professionals recognise and reflect on patients’ circumstances

**DOI:** 10.1177/26323524231164162

**Published:** 2023-03-31

**Authors:** Sam Quinn, Naomi Richards, Merryn Gott

**Affiliations:** End of Life Studies Group, School of Interdisciplinary Studies, Dumfries Campus, University of Glasgow, Dumfries DG1 4ZL, UK; End of Life Studies Group, School of Interdisciplinary Studies, Dumfries Campus, University of Glasgow, Dumfries, UK; Te Ārai Research Group – Palliative Care & End of Life, School of Nursing, University of Auckland, Auckland, New Zealand

**Keywords:** Cross-sector, deprivation, dying at home, end of life, financial insecurity, health, palliative care, poverty, social care

## Abstract

**Background:**

International palliative care policy often views home as the most desirable location for end-of-life care. However, people living in more deprived areas can worry about dying in poor material circumstances and report more benefits from hospital admission at the end of life. There is increasing recognition of inequities in the experience of palliative care, particularly for people living in more deprived areas. Promoting an equity agenda in palliative care means building healthcare professionals’ capacity to respond to the social determinants of health when working with patients near the end of their life.

**Objectives:**

The purpose of this article is to present data which reveal how some health and social care professionals view home dying for people experiencing financial hardship and deprivation.

**Design:**

This work was framed by social constructionist epistemology.

**Methods:**

Semi-structured qualitative interviews (*n* = 12) were conducted with health and social care professionals who support people at the end of life. Participants were recruited from one rural and one urban health board area in Scotland, UK. Data collection occurred between February and October 2021.

**Analysis:**

Thematic analysis was used to analyse the interview data.

**Discussion:**

Our findings suggest that healthcare staff relied on physical clues in the home environment to identify if people were experiencing financial hardship, found discussions around poverty challenging and lacked awareness of how inequities intersect at the end of life. Health professionals undertook ‘placing’ work to try and make the home environment a suitable space for dying, but some barriers were seen as insurmountable. There was recognition that increased partnership working and education could improve patient experiences. We argue further research is needed to capture the perspectives of individuals with direct lived experience of end-of-life care and financial hardship.

## Background

There is increasing recognition that structural inequities pervade palliative care policy and practice and ultimately result in service delivery models that privilege a ‘certain kind of patient’.^1^ While evidence is limited, inequities in access to and experience of palliative care appear to operate along the same social divisions as other forms of privilege, including ethnicity,^2–4^ gender,^5^ age,^6,7^ disability status^8^ and access to financial resources,^9,10^ and the intersections between these. Tackling structural inequities has recently been identified as a global priority for palliative care.^11^ This is further enshrined in the United Nation’s 2030 Sustainable Development Goals (3.8), aiming to achieve universal health coverage including end-of-life care, regardless of income.^12^ However, this work is complicated by the fact that many of these inequities are as invisible as they are pervasive.^1^

One inequity hidden at the heart of most palliative care policy internationally is the importance afforded to the home as a place of end-of-life care and death.^13,14^ This has translated into the prioritisation of community palliative care services, in policy terms at least although this does not always translate to financial investment.^14,15^ In some countries, including the United Kingdom, routinely recording a person’s preferred place of death is part of quality improvement audit processes aimed at increasing the rates of home deaths.^16^ Supporting death at home is also central to palliative care practice and a recent UK study identified palliative care professionals’ view that making places suitable for dying was a key part of their role.^17^ However, what this means when working with populations who live in poor material conditions is unclear. Indeed, palliative care policy envisages a middle-class notion of home as a warm, comfortable and safe space. The limited research conducted in this area indicates that people experiencing financial deprivation and hardship can worry about dying in poor material circumstances^18^ and that those living in more deprived areas report more benefit from a hospital admission at the end of life than do those living in more affluent areas.^19^ These findings are consistent with evidence of an association between lower socioeconomic status and increased likelihood of dying in hospital in several countries.^20^

Promoting an equity agenda within palliative care requires education, training and support for healthcare professionals. Stajduhar *et al.*,^15^ for example, argue that health professionals require enhanced capacity to acknowledge and consider the impacts of the social determinants of health in their provision of end-of-life care, and the importance of developing trust, respect and dignity when working with particular population groups. A recent critical review in this area supported and extended this conclusion by questioning the nature of the support required.^10^ A key gap in current knowledge is the extent to which palliative care professionals currently consider, and potentially respond to, the social determinants of health when working with patients experiencing structural disadvantage. Professional, family and researcher unease around discussing financial issues at the end of life may have contributed to the dearth of research on this topic.^12,21^ Limited research indicates that palliative care professionals recognise financial hardship and deprivation as contributing to the complexity of palliative care needs, but experience barriers to raising the issue of money in conversation with patients.^22^ Bereaved family carers report that they would like to know what additional financial supports are available to them, but health professionals do not discuss this with patients.^13^ The complexity of navigating welfare benefits systems was also highlighted in a recent Australian study which concluded that ‘palliative care clinicians must . . .perceive social needs related to income support and housing as an essential component(s) of palliative care’.^23^ This is in line with recognising ‘financial pain’ as contributing to ‘total pain’, the alleviation of which lies at the centre of palliative care philosophy.^24^ However, the extent to which this translates from policy and underlying philosophy to practice remains unclear, particularly in relation to supporting home dying.

It is within this context that our article explores professionals’ understanding of home dying for people experiencing financial hardship and deprivation.

## Aim and objectives

Interviews were conducted with health and social care professionals as part of a broader study aiming to examine barriers to, and experiences of, home dying for people experiencing financial hardship and deprivation in the United Kingdom called Dying in the Margins.

The aim of this article is to present data which reveal how some health and social care professionals view home dying for people experiencing financial hardship and deprivation.

The following objectives guided this article:

- To identify what characteristics health and social care professionals view as signifiers of a patient experiencing financial hardship;
- To explore professional understanding of the characteristics of patients experiencing financial hardship, including the intersection of different characteristics which might compound inequities experienced;
- To analyse how health and social care professionals envisage their role in supporting home dying for people experiencing financial hardship and deprivation and identify key factors that shape practice.

## Material and methods

This work was framed by social constructionist epistemology.^25,26^ Semi-structured interviews took place between February and October 2021. An interview schedule was prepared beforehand by the research team, but the interviewer was given the freedom to explore interesting tangents that arose during the conversations.^26^ The interview questions (Appendix 1) were developed through analysis of existing literature^10^ and covered the following topics: the main challenges faced by people experiencing financial hardship at the end of life; barriers to accessing home dying for this population; the level of support provided by the state to facilitate home dying where desired and how the COVID-19 pandemic had impacted on current practice compared to pre-pandemic practice.

### Sampling and participants

Overall, 12 professionals who provide support for people at the end of life were interviewed. Participants were identified through convenience sampling, drawn from contacts made by the research team as part of recruitment for the Dying in the Margins study. Interviews were open to health and social care practitioners, along with third sector practitioners, involved in the care and support of people living with serious advanced illness. Informed consent was collected from all participants prior to interview.

One-to-one interviews were conducted with specialist nurses,^4^ a palliative care consultant,^1^ a general practitioner (GP) registrar,^1^ a community link practitioner (CLP),^1^ a social sector practitioner for a cancer information service^1^ and a welfare benefits coordinator.^1^ An additional group interview was conducted with social care practitioners with a role supporting carers.^3^ It should be noted that the group interview was conducted as a matter of convenience to the participants. These data were analysed using the thematic approach used for the other interviews.

Hospice staff were recruited from the central belt of Scotland, as was the CLP. In Scotland, CLPs work within GP practices to provide non-medical support for social and financial issues.^27^ The social care practitioners and a community nurse were recruited from Southern Scotland. A detailed breakdown of participants is presented in Table 1.

**Table 1.** Characteristics of participants.

No.	Organisation	Job title	Area	Gender
1	National Cancer Charity Cancer Information and Support	Support manager	Rural	F
2	Hospice	Palliative medicine doctor	Urban	M
3	Hospice	Palliative medicine doctor	Urban	F
4	Citizens Advice	Welfare benefits coordinator	Rural	F
5	National charity for people with a terminal illness	Nurse – rapid response team	Rural	F
6	National Cancer Charity	Palliative care nurse specialist	Rural	F
7	National charity for people with a terminal illness	Band 5 community nurse	Urban	F
8	Health and Social Care Alliance Scotland	Senior community links practitioner	Urban	F
9	National charity for people with a terminal illness	Senior palliative care nurse	Rural	F
10	[Redacted] Carers Centre	Adult carer support worker (x3)	Rural	F (3)

### Analysis and reporting

A reflexive thematic approach was used in the analysis of the qualitative interview data. Braun and Clarke’s^28^ six-stage process was used to organise data into meaningful themes. This consisted of (1) familiarisation with the interview transcripts; (2) initial coding; (3) identifying themes; (4) reviewing themes; (5) defining themes and (6) writing up. S.Q. and M.G. read through all transcripts before collaboratively developing a coding framework informed by the data. This was then applied by S.Q. to all transcripts. M.G. and S.Q. then developed the themes iteratively through discussion and writing. Theme development was inductive from the data but also shaped by the findings from a critical review of the palliative care and deprivation literature recently led by M.G.^10^ Our analysis was sensitive to the ways in which participants positioned themselves within their interviews and we were attentive to the diversity of views and experiences, particularly between health and social care professionals.

## Findings

The findings are presented in relation to the key themes identified: (1) professionals’ understandings of signifiers of deprivation; (2) characteristics of patients experiencing financial hardship; (3) professional reflections on supporting home dying for people experiencing financial hardship and (4) professional views of improving capacity to support people to die at home through partnership working and education. We present quotations to support each theme. While we provide a job description for each participant, we have chosen not to provide additional socio-demographic information as this could compromise participant anonymity.

### ‘There might be a chair and a television and a bed’: professionals’ identification of signifiers of deprivation

Health professionals used the physical environment of a patient’s home to decipher their financial circumstances. They felt that initiating conversation directly asking them about their financial circumstances could invoke shame for people and therefore they would instead look for clues to indicate that people were struggling financially when doing home visits. As one nurse reflected:

Some people are proud. Some people don’t want to actually voice that they are struggling. I suppose it’s about . . . how we maybe broach that and how we find out what their needs are in the most respectful way . . . I suppose we notice that they’re not using their heating or they’re not actually using their fuel sources and maybe that questioning as to why. (Senior Palliative Care Nurse)

Participants reported that some people would initially deny experiencing financial difficulties, even if the physical environment indicated this might be the case:

People will tell you, ‘No, I’m absolutely fine’. They don’t want to bother you. They don’t want to cause a fuss but actually it’s about being aware and digging a bit deeper. (Senior Palliative Care Nurse)

A cold, unheated house and lack of possessions on display were raised by some participants as signifiers of deprivation and financial hardship:

There might be a chair and a television and a bed. Most houses have a television, but very little in the way of other furniture or books or kitchen appliances and things like that. Sometimes quite cold, quite often quite dirty. (Palliative Care Community Nurse)

Some participants also felt that not being able to keep clothing and household items, such as towels clean, were important markers to look for:

Bars of old soap, that you’re trying to give personal care but they don’t have a clean towel or they’ve only got towels with holes in because they haven’t managed to buy towels. (Rapid Response Team Nurse)

The inability to keep the home clean due to the cost of cleaning products was also seen to extend to personal appearance, with people looking ‘unkempt’ and having dirty clothing because they could not afford to have a washing machine or to buy new clothes. Similarly, not having food in the house was seen to be indicative of a person’s ability to manage financially:

Unkempt, you know, not looking after themselves, very frail, food that’s been in the fridge for a long, long time, it’s all out of date. There’s nothing fresh because either they haven’t got to the shop, or they can’t afford to go to the shop or they’re waiting for a charity to deliver food to them. (Rapid Response Team Nurse)

One of the CLPs discussed how they checked what was in the fridge as a springboard for conversations about financial issues:

‘Can I have a wee look in your cupboard and see what food is there?’- I will ask that. Or I’ll say, ‘Would you like a cup of tea?’, so I can see what’s in their fridge. That kind of thing. So, I will ask open-ended things and if they’re not managing, then I will ask the questions ‘What are you spending [money] on? How can we make this better? And where can we cut back to make sure that you have money for the necessities that you need or don’t get into arrears with your gas and electric? (Senior Community Links Practitioner)

Providing additional education addressing stereotypes and attitudes towards financial hardship was seen as important by some participants:

Some of the people we have are known drug users and then when it comes to them needing heavy drugs at the end of life because of their pain relief, because they have quite a tolerance, just that gap between us and the doctors and getting the thing increased can leave people in pain for quite a long time. So, I think possibly maybe we need some healthcare professional education on discrimination and judgement and things like that. (Band 5 Community Nurse)

The nurses we interviewed expressed concern that some people experiencing financial hardship may have ‘fallen through the net’ during the pandemic. This was because when patients opted out of home visits due to infection fears, health professionals no longer had access to patients’ home environments and could therefore lose access to the usual signifiers of material deprivation and financial hardship.

### ‘There are quite big variations’: professionals’ understanding of the characteristics of patients experiencing financial hardship

Healthcare staff reflected on the heterogeneous nature of the population of people experiencing financial hardship at the end of life; this includes individuals in financial difficulty due to the circumstances of their illness, and people who have longer-term experience of financial hardship. As one participant reflected:

I mean, there are quite big variations in the groups of people that I see. I think some people obviously have had very good jobs over the years and saved a bit of money and financially are managing well or *have* managed well and others have just had to rely on benefits but I think you don’t treat anybody differently for that . . . I think the majority of people that I see would probably financially be having difficulty coping. (Palliative Care Nurse Specialist, National Cancer Charity)

When questioned about how different characteristics might intersect to compound disadvantages at the end of life, participants believed there to be few distinctions between the experiences of men and women, although a palliative medicine doctor reflected that men were more likely to be estranged from relatives, while women faced challenges with supporting their families:

We’ve had a wee run of men who are more likely to be estranged from their families through drink and drugs and that kind of thing, and in and out of jail. With women, it’s maybe different, they may be single parents, or they’ve got financial responsibility for other members of the family, and that can be a real worry for them. (Palliative Medicine Doctor)

There was limited recognition among health staff of how financial hardship might intersect with other protected characteristics, such as ethnicity or gender. One CLP discussed the experience of refugees who were likely to have prior experience of physical and sexual abuse. However, among our rurally based participants, there was significant discussion of the added disadvantage people living rurally could experience.

Our interviewees noted that living rurally often incurred long travel distances between patients’ homes and the hospital and that these were difficult to undertake at the end of life and were not adequately covered by patient transport. Some patients resorted to taking taxis if they could afford to, despite the additional cost. However, this was financially prohibitive for some.

The ability to pay for both formal and informal care was seen to be key in determining the feasibility of home dying in rural areas. Both healthcare workers and third sector staff provided examples of times family and neighbours needed to step in to fill a gap in care so that somebody could remain at home while terminally ill:

[Patient] did stay at home because that’s where she wanted to be, but I think without the access to carers, it wouldn’t have been feasible for her to be on her own. (Rapid Response Team Nurse, Marie Curie)

In some rural areas, there were issues not just with affordability but with the supply of carers as sometimes there were not any care agency staff available in the vicinity:

it’s a nightmare because [the care agency] might say, ‘We can provide one visit, but we don’t actually cover that area’. Nobody covers that area [. . .] It has to be family that take over and then they struggle. (Rapid Response Team Nurse, Marie Curie)

### ‘It wasn’t very straightforward’: professionals’ reflections on supporting home dying for people experiencing financial hardship

Participants were asked to reflect on home dying for people experiencing financial hardship. All the issues they raised related to barriers and challenges for this patient group. Stakeholders reported that they had observed a recent increase in home dying among this population. However, this was not seen to be driven by improvements in decision-making at the end of life, but rather by two factors linked to the COVID-19 pandemic – fear of hospitalisation and anxiety about the possibility of dying alone:

Our caseload for people wanting to die at home has grown quite a lot because people don’t want to go into hospital. They’re frightened of going into hospital because of COVID and also then they can’t have visitors and relatives in either. (Rapid Response Team Nurse, Marie Curie)

When trying to support a person to remain at home, key challenges included houses not being able to physically accommodate medical equipment to support home dying due to small size or being inaccessible. For example, one participant reflected on the unique access challenges posed by high-rise flats:

If you’re living in a high-rise flat getting a hospital bed into the property is near impossible, so you might have to explore other options in terms of the equipment that’s available . . . it’s (also) not uncommon to have lifts breaking in a high rise flat, so patients worry about healthcare staff (visiting). (Palliative Medicine Doctor)

Participants also raised the problem of being able to power electrical equipment. Some patients had insufficient plug sockets and the impact on electricity bills could also be significant. As one participant discussed:

. . . a hospital bed with an air mattress needs two power sockets. We’ve had a patient that only had one power socket in the living room and that’s where the hospital bed was and then they also needed an oxygen compressor, which is another plug socket and so it all adds up . . . things that use electricity as well then impact their electrical bills. (Rapid Response Team Nurse)

There was consistent evidence of healthcare professionals acting outside of what they would consider their ‘normal’ role and trying to remedy issues with the physical environment. Examples of this included topping up electricity metres and contacting local housing associations. As one nurse reflected:

A gentleman a couple of years ago who had chronic lung disease but also a diagnosis of lung cancer . . .. English was not his first language and he’d also lived in an incredibly damp house. I mean I went in and the walls were black and I spent a lot of time, once I’d seen him and done those regular visits with him, I spent a lot of time trying to get onto the Housing Association to try and improve his living accommodation . . . It wasn’t straight forward. I wouldn’t say they were particularly forthcoming in helping and unfortunately, he ended up going into hospital and staying there for end of life care. (Palliative Care Nurse Specialist, National Cancer Charity)

Similarly, a palliative medicine doctor reflected on the need to organise professional cleaning of patients’ homes as part of discharge planning for people experiencing financial hardship. It was particularly evident in the accounts of palliative care professionals that helping to create a nice (or nicer) home environment was seen as a key responsibility to enable home dying.

Directing patients to available financial support was also identified by all participants as a key responsibility for health and social care staff. More straightforward applications for financial support were, in the main, handled directly by healthcare professionals. For additional or more complex benefits, patients were referred to social care professionals, such as a social worker, CLP or the Citizens Advice Bureau. In many cases, emergency grants were used to purchase basic equipment to make the home environment suitable for patients as their condition progressed:

[Cancer Charity], they do one-off grants up to about £600, and I think, again, it’s the nurse specialists who fill them in, but I would expect, more often than not, they’re filling them out for people just to get a washing machine or something that’s a fairly basic, essential piece of life equipment . . . or they’ve not got a bed that’s good enough for them to sleep in, they’re sleeping on a sofa. (Palliative Medicine Doctor)

Participants identified that delays in the receipt of grants for food, clothing and heating were common and some patients would die before these had been processed. As one participant noted:

The benefits take far too long to get. . .[it] is an absolute nightmare, it can take months and months and it is not enough. (Social Care Practitioner)

### ‘The health professionals may not even be aware of some of the other things that are out there’: professionals’ views on improving capacity to support people to die at home through partnership working and education

When asked how to improve end-of-life experiences of people experiencing financial hardship, participants’ responses fell into two broad themes: partnership working and education. As a support manager at a cancer charity commented:

We have a very good working relationship with the Benefits Team at the Citizens’ Advice Bureau so I tend to just directly signpost people to them because they know what’s out there.

Other participants identified barriers to joint working including the inability to easily share information between health services, benefits teams and the third sector:

We don’t use the same systems. The NHS obviously use slightly different systems to perhaps what other third sector, for the likes of ourselves. But we use different systems, we can’t access details or data. Obviously, there’s data protection. I suppose sometimes there’s not always that communication between teams. (Senior Palliative Care Nurse)

It is also notable that in the group interview of adult carer support workers, third sector staff felt that they were not seen as equal partners within multidisciplinary teams. It was suggested that pre-existing notions about certain sectors or organisations could damage partnership working:

We’re a third sector organisation. And I think we’re meant to be equal partners and whatever, but sometimes it doesn’t feel like that. (Social Care Practitioner)

However, it was clear that social care professionals had more experience in working with people experiencing financial hardship and deprivation and had knowledge and expertise that could and did benefit health professionals.

There was notable tension when discussing the issue of education for healthcare professionals and patients. Healthcare participants tended to place the focus on the patient’s personal responsibility to overcome inequities in care, including through education:

It’s the support as well, and I guess just these life skills that I guess, at school and secondary school teaching about money, teaching them about basic things. And I mean, maybe they do that in secondary school, it may be in primary. But basic things about rent, about mortgages. Just these life skills for young people to understand it, so at the time, they get older, they’ve got some idea about managing their money. (Palliative Medicine Doctor)

Healthcare participants also reported an urgent need to upskill people to successfully navigate the health and social care system at the end of life, so that they can make fully informed choices. In particular, they felt further support was required for people who fall into financial hardship when diagnosed with a terminal illness as they were not comfortable navigating the benefits system in the way that people with experience of persistent financial hardship usually are:

Those that generally have been on benefits for a number of years will know that system but those people that maybe are new to benefits because of their health conditions might not know. (Community Link Practitioner)

Education and upskilling of the health and social care workforces on issues affecting people experiencing deprivation and struggling to get by on low incomes were seen as central to improving access to information for patients. Recommendations focused on improving the ability of healthcare staff to signpost to available support and communicate clearly around end-of-life decision-making:

They may well refer to a Benefits Team for more well-known or better-known benefits say Attendance Allowance or PIP (Personal Independence Payment) but they themselves, the health professionals, may not even be aware of some of the other things that are there. So even education for Health and Social Care professionals who are maybe accessing these teams would be helpful too. (Support Manager)

## Discussion

Our data provide new insights into how health and social care professionals view home dying for people experiencing financial hardship and deprivation. A key finding was that healthcare professionals supporting those at the end of life tended to use the physical environment as an indicator of financial circumstances, reporting that patients can be unwilling to discuss financial concerns they are experiencing. Richards^29^ highlights how, despite the equity-driven agenda developing in palliative care, current language around financial hardship remains stigmatising, and a source of difficulty for healthcare professionals. Our findings highlight the need for strategies and tools to aid health professionals in facilitating conversations on financial hardship and deprivation. There are risks in overlooking people who are experiencing financial hardship if professionals are solely relying on signifiers in the material environment. Moreover, the nature of the signifiers health professionals looked for, for example, a dirty house, could themselves be seen as conforming to a stigmatising stereotype. Participants with more experience in supporting people living with financial hardship (CLP, Benefits Coordinator) offered examples of strategies to help facilitate conversations on financial issues, such as exhibiting a non-judgmental attitude, asking open-ended questions and practicing deep listening.^30^

Most participants did not identify compounding intersecting inequities, such as gender and ethnicity. This almost certainly reflects a lack of awareness of how intersectional inequities can shape end-of-life experiences rather than that people were not experiencing multiple or compounding disadvantages at the end of life.^15^ Indeed, while research is very limited in this area, there is considerable evidence to support the need for an intersectional lens when understanding health inequities.^5,31,32^ Participants *did* note a difference between end-of-life experiences for people living in urban and rural areas, and the impact on financial hardship. In particular, travel costs were reported to be high for patients living rurally because of the distances involved and due to the limited availability and capacity of patient transport – costly taxis had to be used^33^ In line with previous research,^34^ having support from family and paid carers was seen as absolutely key to facilitating home dying in rural areas. Paying for care was identified as particularly challenging for people experiencing financial hardship.

Our findings support previous evidence regarding the role of the physical environment of the home in limiting opportunities for dying at home.^35^ Limited space to accommodate medical equipment and access challenges within tower blocks and tenements were identified as key barriers. Our findings confirmed those of Driessen *et al.*^17^ that palliative healthcare staff try to continually adjust the physical environment of the home to make it suitable for dying, activities Driessen *et al.*^17^ define as ‘placing work’. There were notable examples of this, with healthcare staff stepping outside of what could be considered their professional role, for example, organising cleaners, and topping up electricity meters to enable patients to be comfortable in their home environment. This extra ‘placing work’ makes sense considering the importance that palliative care professionals attribute to home dying, sometimes to the extent that if a patient does not die at home, they can view this as ‘falling short of their duties’.^17^ Further research could usefully explore the experiences of patients and families who are on the receiving end of such ‘placing work’, including whether those experiencing financial hardship share the same understanding as healthcare professionals regarding what constitutes a suitable home environment for end-of-life care.

There was some acknowledgement among participants that palliative care professionals could stand to benefit from closer partnership working with professionals who have a clearer focus on addressing the social determinants of health. However, social sector staff raised concerns they were not viewed as equal partners by healthcare professionals, a view that is not uncommon.^36^ This reveals a symbolic recognition gap between health and social care professionals: healthcare professionals perceived^37^ that they were stepping outside of their role, while social care professionals felt that they were not viewed equally by their healthcare counterparts. There is therefore a need to support partnership working between health and social care professionals in providing equitable care to structurally marginalised populations and to increase awareness among both groups of the nature and unique contribution that the other speciality makes within this context.

There was a view among many participants working in both the health and social care sectors that they could benefit from education to enhance their understanding of issues affecting people experiencing deprivation. Qualitative interviews with 24 GPs in some of Scotland’s most socio-economically disadvantaged areas found that only GPs fluent in discussing the structural causes of health inequalities felt an obligation to change local systems and strengthen community services. It was found that while some GPs working in disadvantaged areas had structural competency, there was still scope to broaden the understanding of the structural determinants of health^38^ Our findings indicate that a key area where such initiatives should focus is supporting health professionals to recognise the structural and intersecting nature of inequities. We identified that there are still elements of a ‘blame’ culture^39^ which exist and a focus on individual responsibility as a means of overcoming inequities in care rather than on structural change.

Many health systems are moving towards health and social care intermediaries as a way of addressing the social determinants of health; for example, the expansion of the CLP programme in both England and Scotland.^40,41^ These intermediaries operate as non-clinical social prescribers, providing support for issues beyond the scope of medical treatment.^27^ Our findings indicate that intermediaries/social prescribers appear to play an important role in supporting end-of-life care at home for people experiencing financial hardship and deprivation, although more research is needed.

While remaining at home at the end of life is generally viewed as a desirable outcome, this is not necessarily true for people experiencing financial hardship and deprivation. As highlighted by Marie Curie’s 2022 Dying in Poverty report,^42^ home dying can involve a number of inescapable costs, such as childcare and high energy bills, which means it is not always accessible to all. Given rising levels of poverty around the world,^43^ further research is urgently required to understand better what kind of initiatives can support end-of-life care at home for people struggling to make ends meet. A crucial evidence gap to fill relates to the experiences and preferences of individuals and their families receiving end-of-life care at home.^10^ Strengths-based participatory research is needed that values the expertise of individuals with lived experience of poverty and their wider social group, and the health and social care sector practitioners who aim to support them.

## Recommendations for policy and practice

Based on our findings and analysis, we make the following recommendations:

- Undertake work to facilitate more open conversations about financial hardship and deprivation at the end of life among health and social care professionals.
- Adopt a place-based approach to tackling the financial costs associated with a terminal illness; particularly fuel poverty and transport costs, reflecting the different needs in rural and urban areas.
- Simplify access to welfare and benefits support for people with a life-limiting condition and their carers. For example, by including financial considerations as part of a needs assessment or care plan.
- Further research is needed to understand the role of the home environment in shaping the end-of-life experiences of people experiencing financial hardship. Such work may also consider the role of social housing providers in end-of-life care.
- A whole system approach is needed to improve the financial security of terminally ill patients and carers, this includes opportunities for joint learning and training around financial hardship and deprivation for all health and social care staff supporting people with a terminal illness.

## Strengths and limitations

The limitations of this study should be noted to provide context for the interpretation of the results. Data collection was conducted in two Scottish health boards and this context should be remembered when considering the applicability of these findings to other geographical areas. In addition, the interviews were conducted during the COVID-19 pandemic. We believe there would be value in future research exploring professional’s experiences in this area given the post-pandemic economic difficulties faced in many countries.^44^

We have presented a cross-sector account of professional views on home dying for people experiencing financial hardship and deprivation which broadens our understanding of how health professionals identify, communicate and perform their role within this context. We have also started to address the dearth of evidence regarding access to home dying for people experiencing poverty and deprivation in rural areas.^45^ We acknowledge a gap in terms of capturing the firsthand experiences of those with lived experience of end of life and financial hardship and, within our discussion, argue for these perspectives to be better represented in future research.

## Conclusion

With the growing equity focus in palliative care, we have explored health and social care professionals’ views on home dying for people experiencing financial hardship. Our findings suggest that healthcare professionals face difficulty discussing patients’ finances with them, preferring instead to use the home environment to make an implicit assessment of their financial circumstances. Higher transport costs and the greater challenge of accessing formal care were seen to be key determinants in shaping end-of-life experiences in rural areas. An unsuitable home environment was considered by our participants as a primary barrier to facilitating home dying for people experiencing financial hardship, regardless of urban/rural location. Health professionals consistently undertook actions to make the physical environment more suitable for dying – in their eyes. Participants identified that cross-sector partnership working could improve services; however, those working in the social sector felt that they were not always seen as equal partners. The need to educate healthcare professionals was discussed, although there was some focus on individual responsibility as a means of overcoming inequities in care, indicating elements of a ‘blame’ culture. Finally, we argue that patient and carer perspectives are currently underrepresented in the academic evidence base. We call for more participatory research which values the expertise of individuals with lived experience of end-of-life care and financial hardship.

## Supplemental Material

sj-docx-1-pcr-10.1177_26323524231164162 – Supplemental material for Dying at home for people experiencing financial hardship and deprivation: How health and social care professionals recognise and reflect on patients’ circumstances Click here for additional data file. Supplemental material, sj-docx-1-pcr-10.1177_26323524231164162 for Dying at home for people experiencing financial hardship and deprivation: How health and social care professionals recognise and reflect on patients’ circumstances by Sam Quinn, Naomi Richards and Merryn Gott in Palliative Care and Social Practice

## Declarations

**Ethics approval and consent to participate:** This research was approved by the University of Glasgow College of Social Sciences Research Ethics Committee (ID: 400190195). Written informed consent to participate was obtained from participants.

**Consent for publication:** Written informed consent for publication was provided by all participants.

**Availability of data and materials:** Data available on request due to privacy/ethical restrictions.
