# Supplementary material for: Dying at home for people experiencing financial hardship and deprivation: How health and social care professionals recognise and reflect on patients’ circumstances
Source: Palliat Care Soc Pract. 2023 Mar 31;17:26323524231164162. doi: 10.1177/26323524231164162 (PMC10071150; doi:10.1177/26323524231164162)
Supplement: sj-docx-1-pcr-10.1177_26323524231164162 – Supplemental material for Dying at home for people experiencing financial hardship and deprivation: How health and social care professionals recognise and reflect on patients’ circumstances [file sj-docx-1-pcr-10.1177_26323524231164162.docx]

**Appendix 1 – Health and Social Care Professional Interviews Schedule**

Name:

Organisation:

Role:

1. To start with, could you tell me about your professional role?
2. How has the pandemic impacted your practice?
3. In your work, do you encounter people who are:

- struggling to get by on a low income?

- living in unsuitable housing?

4. In your experience, what are the big issues affecting people in such circumstances?

5. Thinking about those people, do you think there are additional costs at the end of life?

6. How important are poverty and deprivation in comparison to say gender or age in accessing services?

7. Are there any issues with the amount or type of care the state will provide at home?

8. Are there additional challenges for people in rural areas?

9. What more could we do to support people affected by poverty at the end of life?

10. Can you talk to me about the choices people living in poverty might make at the end of life around their place of death?

11. Have you encountered people who wished to die at home and then this was not possible?

- What were the challenges?

13. What would you like to tell people who are designing services about how people’s circumstances affect them at this time in their lives?

14. Can you recommend anyone else who works in this area or on these issues who we should speak to?
